# Case Report: Effective use of rituximab in children with steroid-resistant/dependent idiopathic pulmonary hemosiderosis: description of two cases and literature review

**DOI:** 10.3389/fped.2026.1944579

**Published:** 2026-09-28

**Authors:** Sha Li, Tinghua Liu, Rongqiong Ou, Haiyan Wang, Bihong Zhang, Weiping Tan

**Affiliations:** Department of Pediatrics, Sun Yat-sen Memorial Hospital, Sun Yat-sen University, Guangzhou, China

**Keywords:** alveolar hemorrhage, children, rituximab, steroid resistant, steroid-resistant/dependent idiopathic pulmonary hemosiderosis

## Abstract

**Introduction:**

Idiopathic pulmonary hemosiderosis (IPH) is a rare, chronic, and potentially life-threatening disorder predominantly affecting the pediatric population. While most patients respond to conventional immunosuppressive therapy, management of steroid-resistant/dependent cases remains challenging. This study aims to evaluate the efficacy and safety of rituximab (RTX) in the treatment of steroid-resistant/dependent IPH in children.

**Methods:**

We report two pediatric cases of steroid-resistant/dependent IPH who were admitted to our institution. Both patients presented with severe, recurrent diffuse pulmonary hemorrhage and demonstrated poor response to conventional therapy including high-dose corticosteroid pulse therapy and combination immunosuppressants. Clinical data, laboratory findings, radiographic features, treatment regimens, and outcomes were collected and analyzed.

**Results:**

Both patients exhibited steroid resistance or dependence with persistent or recurrent pulmonary hemorrhage despite aggressive conventional immunosuppression. Following RTX administration, rapid and sustained hemorrhage control was achieved in both cases. Clinical remission was maintained over follow-up periods of 6 months for both patients, with successful corticosteroid tapering and no significant infusion-related adverse events observed. No disease relapse occurred during the follow-up period.

**Conclusion:**

Rituximab appears to be an effective and well-tolerated rescue therapy for steroid-resistant/dependent IPH in children, offering sustained hemorrhage control and facilitating corticosteroid-sparing. Given the limited existing literature on RTX application in IPH management, this report offers a novel therapeutic approach for steroid-resistant/dependent IPH cases.

## Introduction

Idiopathic pulmonary hemosiderosis (IPH) is a rare disease of unknown etiology that predominantly affects children and adolescents，with an estimated incidence ranging from 0.24 to 1.26 cases per million children (1). The clinical manifestations are diverse and nonspecific, often presenting with recurrent cough, hemoptysis, and anemia as the main symptoms, leading to frequent misdiagnosis or missed diagnosis. Traditional treatments primarily include corticosteroids and immunosuppressants, which can control the condition in some patients. However, a small subset of steroid-resistant/dependent cases responds poorly, resulting in a high mortality rate, with most of the affected patients ultimately succumbing to pulmonary hemorrhage and respiratory failure. This study presents two pediatric cases of steroid-resistant/dependent IPH.

## Case reports

### Case 1

The first patient was a 16-month-old female who presented with cough and severe anemia (nadir hemoglobin: 45 g/L)，with no clinical manifestations suggestive of celiac disease, such as chronic diarrhea, abdominal distension, or nutritional deficiency. Chest computed tomography (CT) demonstrated bilateral multifocal pulmonary infiltrates. Bronchoscopy revealed a congested bronchial mucosa with copious pale red secretions, while bronchoalveolar lavage (BAL) fluid analysis identified hemosiderin-laden macrophages. Comprehensive microbiological investigations—including bacterial cultures, viral PCR, fungal assays, Mycoplasma pneumoniae serology, and tuberculosis testing in blood, sputum, and BAL fluid—were negative. Immunological workups, including anti-neutrophil cytoplasmic antibodies (ANCA), anti-nuclear antibodies, and anti-glomerular basement membrane antibodies, coagulation studies, and cardiac evaluation yielded unremarkable results；cow's milk protein allergy screening was negative. Lymphocyte subset analysis demonstrated elevated B-cell proportions (62.94%) with markedly increased CD20+ B cells (62.17%), prompting a provisional diagnosis of idiopathic pulmonary hemosiderosis (IPH).

Initial management comprised high-dose intravenous methylprednisolone pulse therapy (10 mg/kg/day for 2 days, followed by 5 mg/kg/day for 3 days), subsequently tapered to 2 mg/kg/day, supplemented with leflunomide and broad-spectrum antibiotics. Despite persistent reticulocytosis, the patient presented with refractory anemia and persistent respiratory symptoms. Considering the rapidly progressive disease course and poor clinical response to corticosteroid monotherapy, excessive B-cell activation was highly suspected, and the patient was therefore administered rituximab (RTX) at a dose of 375 mg/m^2^ in September 2024. Hemoglobin levels returned to normal within two weeks after RTX initiation, enabling gradual corticosteroid tapering.

During the subsequent 5-month follow-up, peripheral CD20⁺ B cells remained persistently undetectable, concurrent with hypogammaglobulinemia characterized by decreased serum IgG levels. Intravenous immunoglobulin (IVIG) was supplemented at a dosage of 0.5 g/kg per administration, administered once monthly for three consecutive times. for three consecutive times. Seven months after the first RTX treatment, the patient developed intestinal infection, which achieved significant improvement after 3 days of empirical antimicrobial therapy. At the 8-month follow-up, flow cytometry showed obvious B-cell reconstitution, with CD19⁺ B cells accounting for 18.7% and CD20⁺ B cells accounting for 18.5%. Meanwhile, chest computed tomography (CT) revealed extensive exudative lesions in both lower lungs. Given the recovery of peripheral B lymphocytes and progressive pulmonary lesions, reduced-dose RTX (178 mg/m^2^) was administered as the second consolidation cycle in May 2025.

Eight months after the second RTX consolidation treatment, repeated chest CT performed in an external hospital indicated recurrent pulmonary exudation. Bronchoalveolar lavage (BAL) detected alveolar hemorrhage, and next-generation sequencing (NGS) of BAL fluid identified multiple pathogens, including cytomegalovirus (CMV), Epstein–Barr virus (EBV) and *Candida albicans*. Flow cytometry revealed further elevated B-cell counts, with CD19⁺ B cells at 19.9% and CD20⁺ B cells at 20.7%. Accordingly, the patient received the third cycle of RTX at 250 mg/m^2^ in January 2026. Methylprednisolone pulse therapy was given at 5 mg/kg/d for three consecutive days, followed by 2 mg/kg/d for an additional three days, then tapered to 1 mg/kg/d for maintenance. Leflunomide was continued as background immunosuppression, whereas hydroxychloroquine was initiated *de novo* during this disease flare, alongside pathogen-directed anti-infective therapy. The patient has been followed up regularly in the outpatient department for six months after the third RTX treatment, and no disease relapse has been observed. Corticosteroids have been further tapered to a maintenance dose of prednisone 2.5 mg once daily，with hemoglobin levels persistently maintained within the normal range during follow-up (Figures 1 and 2).

**Figure 1:**
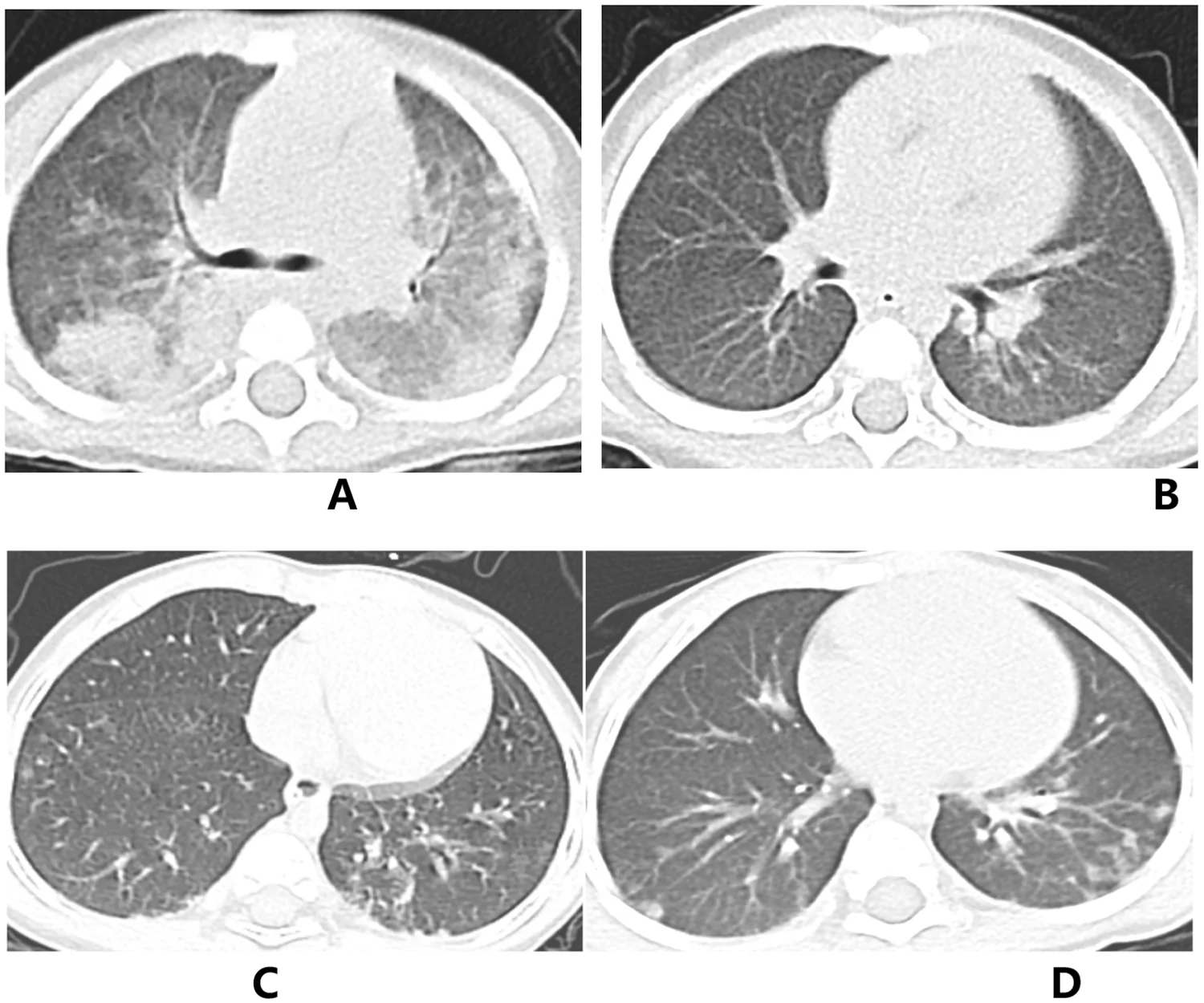
Chest CT scan images during sequential rituximab (RTX) therapy. **(A)** Baseline CT before the first RTX cycle (September 2024). **(B)** CT obtained 3 months after initial RTX therapy (January 2025). **(C)** Pre-treatment CT prior to the second RTX cycle (May 2025). **(D)** Pre-treatment CT prior to the third RTX cycle (November 2025).

**Figure 2:**
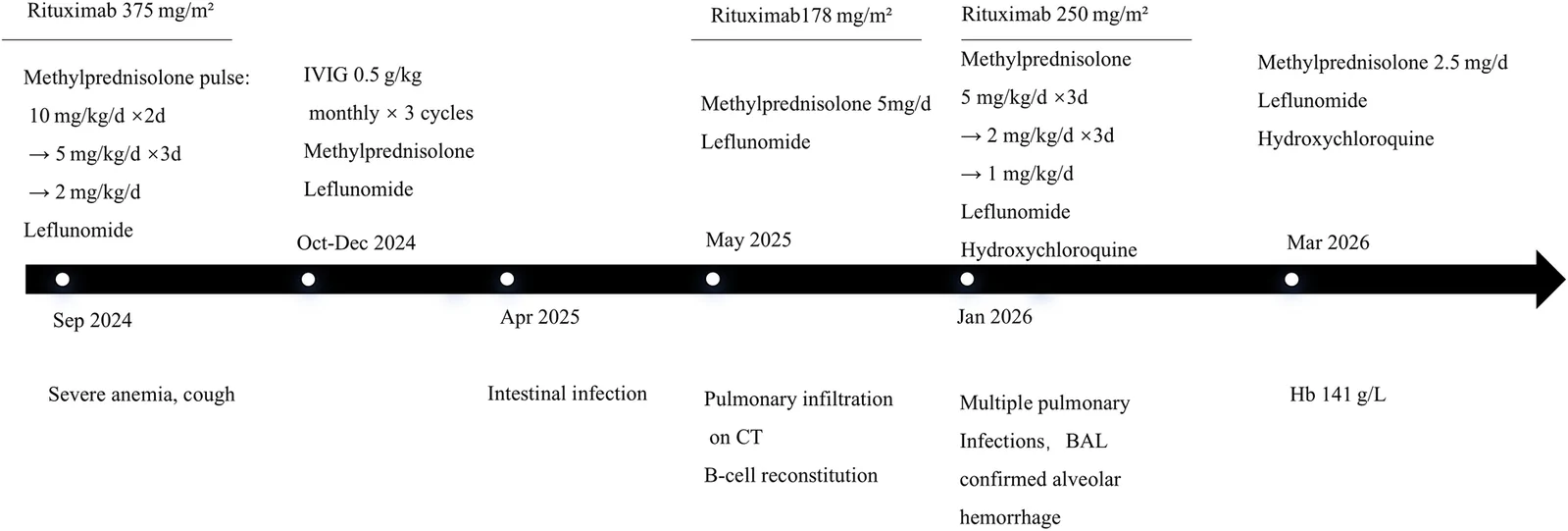
Clinical timeline of disease course and therapeutic interventions for patient 1 with idiopathic pulmonary hemosiderosis.

### Case 2

The second case involved a 12-year-old female patient diagnosed with IPH at 5 years of age (February 2018) following clinical manifestations of severe anemia (hemoglobin: 53 g/L) and concurrent pneumonia，with no celiac disease-related symptoms and a negative cow's milk protein allergy screening result. Diagnostic confirmation was achieved via BAL, which demonstrated abundant hemosiderin-laden macrophages. Initial therapeutic intervention consisted of oral prednisone (10 mg three times daily), with subsequent tapering to 10 mg twice daily after 4 months. The first recurrence of hemoptysis occurred in late July 2018, approximately two weeks after self-discontinuation of prednisone therapy. Treatment was reinstated at the original dosage (10 mg three times daily) and gradually reduced to 10 mg twice daily over a 3-month period. A second hemoptysis episode was documented in November 2018, accompanied by radiographic findings of bilateral patchy ground-glass opacities on chest CT and hemosiderin-laden macrophages on sputum cytology. Consequently, the patient underwent intravenous cyclophosphamide therapy (0.4 g biweekly for a total of 6 doses).

Upon admission to our department in April 2019 (at 6 years of age), laboratory evaluation revealed a hemoglobin level of 95 g/L and elevated Mycoplasma pneumoniae antibody titers (1:160), with no other significant pathogens or immunological abnormalities identified. Immunomodulatory therapy was augmented with leflunomide while prednisone maintenance was continued at 10 mg once daily. Clinical stability was maintained, with hemoglobin levels ranging from 116 to 124 g/L. Due to corticosteroid-related adverse effects, including short stature and obesity, prednisone was systematically tapered over two years to 3.75 mg once daily (initiated March 2021).

Over the subsequent four years, the child experienced three recurrent episodes of hemoptysis, with hemoglobin levels dropping to a nadir of 63 g/L. Multiple bronchoalveolar lavage fluid cytological examinations confirmed hemorrhage and the presence of hemosiderin-laden macrophages. CT imaging revealed diffuse ground-glass opacities and ill-defined pulmonary infiltrates. Culture of bronchoalveolar lavage fluid identified infections with Tropheryma whipplei, Streptococcus pneumoniae, and Pseudomonas aeruginosa. Pulmonary function tests demonstrated moderate obstructive ventilatory dysfunction. The patient received intravenous methylprednisolone pulse therapy (10 mg/kg/day for 3 days), hydroxychloroquine, and antimicrobial treatment, resulting in clinical symptom resolution. However, disease recurrence occurred after hydroxychloroquine discontinuation, when prednisone was tapered to a maintenance dose of 2.5 mg once daily.

In June 2025, the patient experienced a fifth disease recurrence, flow cytometry demonstrated a marked increase in B-cell populations (48.56%, CD20^+^ 47.67%). Given the persistent CD20^+^ B-cell expansion and inadequate disease control, RTX therapy(375 mg/m^2^) and intravenous methylprednisolone(2 mg/kg/day) was administered, resulting in complete CD20^+^ cell depletion (0%) within one week. Post-discharge maintenance therapy consisted of oral prednisone(1 mg/kg/day), leflunomide. Six months later, the patient was readmitted due to cough. Chest CT indicated multiple exudative lesions in the right lung (Figure 3). The levels of CD19+ B cells and CD20+ B cells were 2.96% and 2.8%, respectively. A second course of rituximab was administered intravenously at a dose of 375 mg/m^2^ in December 2025. Over a 6-month follow-up, prednisone was successfully tapered to 7.5 mg once daily without recurrent hemoptysis. By June 2026, hemoglobin levels normalized to 139 g/L (Figure 4).

**Figure 3:**
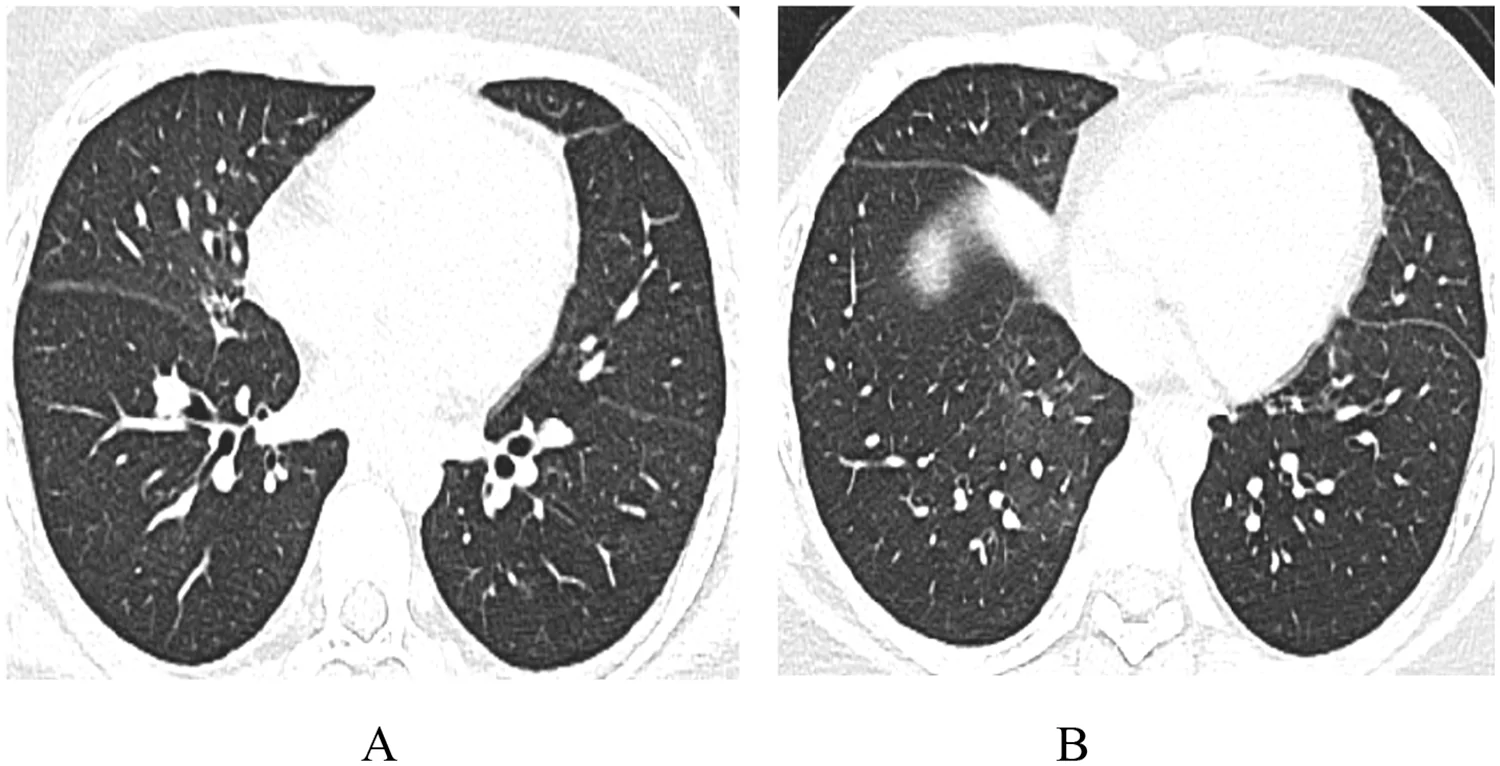
Chest CT scan images obtained in January 2026. **(A,B)** Two different axial CT slices revealing multifocal patchy opacities distributed in the apical segment of the right upper lobe, medial basal segment, lateral basal segment of the right lower lobe, and the right middle lobe.

**Figure 4:**
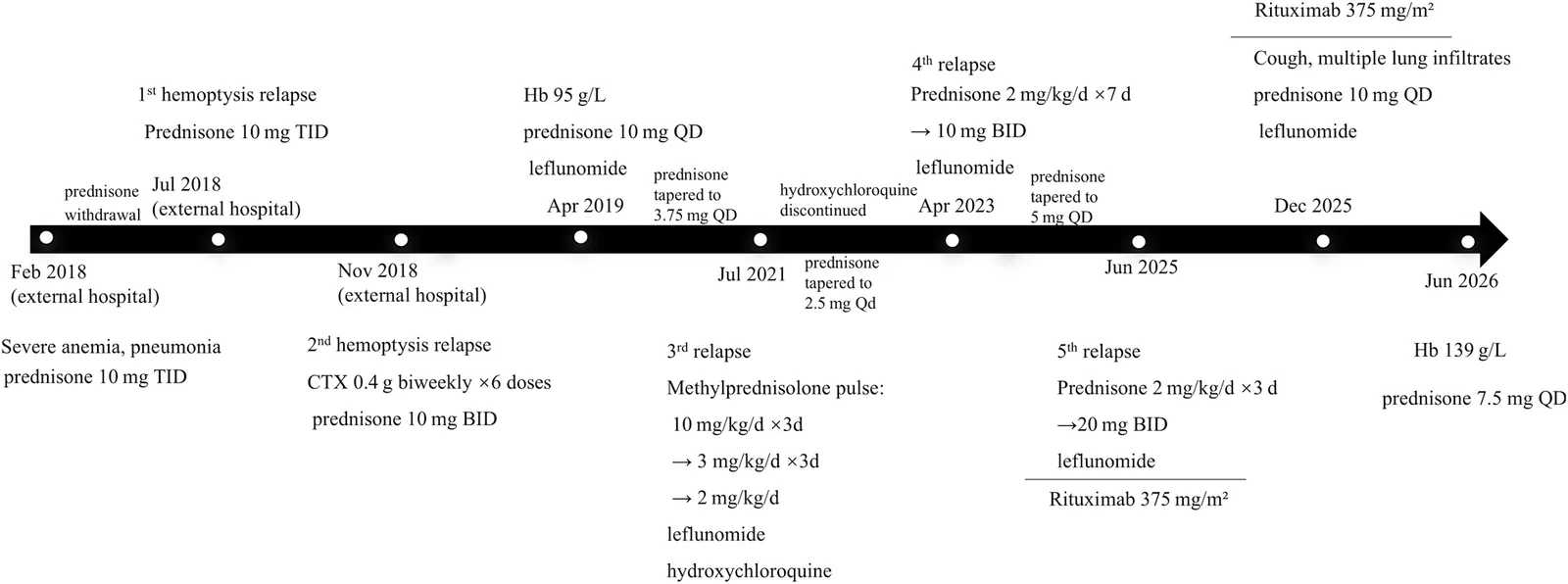
Clinical timeline of disease course and therapeutic interventions for patient 2 with idiopathic pulmonary hemosiderosis. Inter-segment annotations denote medication adjustments occurring between two documented time-points. BID, twice daily; QD, once daily; TID, three times daily; CTX, cyclophosphamide.

## Discussion

IPH is clinically characterized by recurrent episodes of diffuse alveolar hemorrhage, typically manifesting as microcytic hypochromic anemia alongside respiratory symptoms such as cough and hemoptysis. Although its precise etiology remains incompletely understood, growing evidence points to an association with immune dysregulation (2–7). Diagnosis is primarily one of exclusion, and current treatment strategies largely rely on glucocorticoids, often in combination with immunosuppressive agents such as cyclophosphamide, hydroxychloroquine, azathioprine, and mycophenolate mofetil (8–11).

Most children diagnosed with idiopathic pulmonary hemosiderosis (IPH) demonstrate satisfactory clinical responses to conventional immunosuppressive regimens. Nevertheless, a small subset of pediatric patients with steroid-resistant/dependent IPH exhibits suboptimal responses to standard therapeutic approaches. Such patients frequently manifest a protracted disease trajectory, recurrent episodes of alveolar hemorrhage, substantial challenges during glucocorticoid tapering, and progressive impairment of pulmonary function.

Notably, no formal consensus-based treatment guidelines have been developed for this distinct patient population. High-grade clinical evidence regarding optimal medication selection, comparative therapeutic efficacy, and long-term medication safety remains limited.

The present study describes two pediatric cases of steroid-resistant/dependent IPH characterized by acute onset, recurrent symptoms, and inadequate responses to glucocorticoid therapy. Despite intensive treatment with high-dose corticosteroid pulse therapy and combined immunosuppressive regimens, persistent pulmonary hemorrhage was observed in both patients. Elevated peripheral CD20+ B lymphocyte counts prompted the initiation of rituximab (RTX) treatment, which achieved effective and sustained control of alveolar hemorrhage, stable clinical remission, and successful tapering of systemic corticosteroids.

Current evidence on the use of RTX in refractory IPH remains scarce. A systematic search of PubMed was performed using the terms “rituximab” and “idiopathic pulmonary hemosiderosis”. Given that IPH is a diagnosis of exclusion, cases with other potential etiologies for alveolar hemorrhage were strictly excluded. Five English-language publications with definite diagnosis were finally identified, which reported a total of 11 pediatric and one adult case of IPH. These patients received RTX after failing corticosteroid or combined immunosuppressive therapy, with most showing positive clinical outcomes (Table 1).

**Table 1:** Literature review on the use of rituximab for idiopathic pulmonary hemosiderosis.

Author, year published.	Cases	Sex	Country	Age at onset/ diagnosis (years)	Age at RTX initiation (years)	Presenting symptoms	Prior therapies	RTX indication	RTX protocol	Response to RTX	RTX-related side effects	Conclusions
Gripaldo et al, 2021 (12)	1	Female	USA	24/24	24	Hemoptysis, dyspnea	High-dose steroids with a slow taper over several weeks → pulse dose steroids	Massive hemoptysis and severe hypoxemia during steroid tapering	1 gram once weekly for 2 weeks	Symptoms resolved promptly. Remission sustained at 6 months	NS	Safe and effective alternative to cyclophosphamide for DAH in IPH
Argraves et al, 2022 (13)	7	Male/ Female: 2/5	USA	NS	Median: 6.81 [IQR: 6.10, 10.41]	Anemia, cough, hemoptysis, dyspnea, etc.	Corticosteroids and steroid-sparing agents (AZA, IVIg, etc.)	Steroid-sparing effects	NS	At 6 months: Improved Hb, no hospitalizations, 14% (1/7) off steroids	NS	Demonstrated steroid- and hospitalization-sparing effects at 6 months
Terheggen-Lagro et al., 2022 (14)	2	Male; Female	The Netherlands	neonatal period/4; 4/6	9; 12	Patient 1: Recurrent dyspnea with severe anemia; Patient 2: Anemia, pneumonia	Patient 1: Frequent systemic steroid pulse therapy + HCQ + MMF for 5 years; Patient 2: Systemic corticosteroids for 6 years， including intermittent methylprednisolone pulse therapy + HCQ + MMF for 5 years	Patient 1: Declining lung function and steroid-related toxicity; Patient 2: Recurrent hemorrhage and steroid-related toxicity	Every 6 months for ≥ 4 years (dose NS)	Patient 1: Off prednisolone at 15 months, improved lung CT/ function; Patient 2: Off prednisolone at 9 months, improved lung CT/function, fewer exacerbations, and better compliance	Patient 1: Mild hypogammaglobulinemia; Patient 2: none	Effective, safe, steroid-sparing for long-term pediatric IPH
Louey et al, 2025 (15)	1	Female	Australia	4/4	11	Hemoptysis, anemia	Intravenous monthly methyl-prednisolone 15 mg/kg/day for 3 doses → AZA (for 7 years) → systemic corticosteroids + MMF after recurrence	Severe hemorrhage and steroid side effects	375 mg/m^2^ weekly for 2 doses, with a third dose at 6 months	Corticosteroids ceased; clinically well, and returned to school for the first time in 12 months	NS	Can successfully induce remission in pediatric IPH
Aldaithan et al, 2026 (16)	1	Female	Kuwait	7	7	Recurrent hypoxemic episodes, diffuse alveolar bleeding, severe anemia	Corticosteroids, hydroxychloroquine, azathioprine	Steroid-dependent IPH, relapses upon steroid reduction	375 mg/m^2^ weekly for 4 weeks	Significant clinical improvement, durable remission, off steroids and other immunosuppressants	NS	Rituximab is a promising steroid-sparing option for refractory pediatric IPH

NS, Not Specified; IPH, idiopathic pulmonary hemosiderosis; DAH, diffuse alveolar hemorrhage; RTX, rituximab; HCQ, hydroxychloroquine; AZA, azathioprine; CTX, cyclophosphamide; IVIG, intravenous immunoglobulin; MMF, mycophenolate mofetil.

Regarding its pathogenesis, several hypotheses have been proposed for IPH, the most prominent theory involves immune dysregulation (11). Studies have detected nonspecific autoantibodies—such as ANCA, anti-alveolar basement membrane antibodies, and anti-nuclear antibodies—in the serum of some patients with IPH, likely derived from activated B cells (17). Notably, a recent study reported a 26.4% overall autoantibody positive rate in pediatric IPH, including celiac disease-related antibodies (tTG-IgA, EMA-IgA, DGP) (25.9%), SMA (23.2%), ANA (20.3%), ANCA (17%), RF (12%), and anti-dsDNA antibodies (9.1%) (18). IPH has also been associated with cow's milk protein allergy. In our cohort, both patients tested negative for cow's milk protein allergy; celiac disease serology was not performed due to absent clinical symptoms. As an immunosuppressant, RTX is a chimeric IgG1 monoclonal antibody that targets CD20 on B lymphocytes, leading to B-cell depletion and immunomodulation (11). Its reported efficacy in certain patients with IPH in recent years further supports a potential pathogenic role for B cells (14). In the two pediatric cases we reported, significantly elevated B-cell counts were observed, and disease control was achieved following RTX treatment, suggesting that disease development in such children may be linked to B-cell activation. However, the precise mechanisms remain unclear and warrant further basic and clinical investigation.

Importantly, some children initially diagnosed with IPH may subsequently develop AAV or other autoimmune diseases, highlighting the immunological overlap and the need for long-term follow-up.

RTX has demonstrated efficacy in various immune-mediated rheumatic diseases, including ANCA-associated vasculitis (AAV) (19), rheumatoid arthritis (RA) (20), systemic sclerosis (21), and lupus nephritis (22). Several published case series and case reports have described favorable clinical responses to RTX among patients with refractory IPH [12-16]. Although cyclophosphamide has historically been used in steroid-refractory IPH, RTX may represent a safer alternative (11). Currently, no standardized RTX dosing regimen exists for IPH, though protocols from RA or AAV may serve as references. In RA, the typical regimen is 1 g administered in two divided doses two weeks apart (23). In contrast, rituximab dosing for pediatric anti-neutrophil cytoplasmic antibody-associated vasculitis (AAV) generally adheres to the regimen outlined in the RAVE trial: 375 mg/m^2^ administered once weekly for four consecutive weeks (24). In the present study, Case 1 received an initial RTX dose of 375 mg/m^2^, followed by a reduced dose of 178 mg/m^2^ eight months after the first infusion, and a third dose of 250 mg/m^2^ seven months after the second infusion. Case 2 was administered an initial dose of 375 mg/m^2^, with a second identical 375 mg/m^2^ infusion delivered six months later. Both patients responded to doses lower than those commonly reported, with no recurrence observed during follow-up.

The safety profile of RTX in immune-mediated diseases is generally favorable. The first three months after infusion carry the highest risk of infections (incidence 30%) (25), hypogammaglobulinemia (16%) (26), and infusion-related reactions (41%) (27). In Case 1 of this study, IgG levels declined one month after RTX initiation, prompting prophylactic IVIG supplementation (0.5 g/kg monthly for three doses) to prevent infection. This patient developed an intestinal infection seven months after the first RTX infusion, which resolved with standard anti-infective therapy. Following a 50% dose reduction during the second RTX course, the patient experienced a mild pulmonary infection. No further adverse effects were noted from the third RTX dose through the present follow-up. Case 2 showed no significant adverse reactions throughout the follow-up duration after RTX treatment.

In summary, IPH exhibits considerable heterogeneity in immunopathological mechanisms and treatment responses. For steroid-resistant/dependent cases with features of immune dysregulation—particularly those with elevated B-cell counts—RTX may represent a promising therapeutic option. However, this study included only two patients with a relatively short follow-up duration of 6 months. Although short-term remission was achieved, long-term efficacy, relapse risk and safety cannot be fully assessed. Further clinical trials are needed to establish patient selection criteria, determine optimal dosing regimens (including timing, dosage, and duration), and evaluate the long-term prognostic impact of RTX in steroid-resistant/dependent IPH.

## Data Availability

The datasets presented in this article are not readily available because the raw clinical data cannot be publicly shared to protect the patient’s privacy. Data may be available from the corresponding author upon reasonable request after appropriate ethical review. Requests to access these datasets should be directed to Sha Li, lisha36@mail.sysu.edu.cn.
